# Advancing a cultural change agenda in higher education: issues and values related to reimagining academic leadership

**DOI:** 10.1007/s43621-022-00079-6

**Published:** 2022-03-30

**Authors:** Joseph A. Whittaker, Beronda L. Montgomery

**Affiliations:** 1 Division of Research and Economic Development, Jackson State University, Jackson, MS 39217 USA; 2 DOE-Plant Research Laboratory, Michigan State University, East Lansing, MI 48824 USA; 3 Department of Biochemistry and Molecular Biology, Michigan State University, East Lansing, MI 48824 USA; 4 Department of Microbiology & Molecular Genetics, Michigan State University, East Lansing, MI 48824 USA

**Keywords:** Educational ecosystems, Higher education, Evidence-based innovation, Institutional transformation, Leadership

## Abstract

Traditional models of academic leadership are based largely on managerial and transactional approaches. Such efforts frequently support status quo individual success rather than values-based leadership based on collective institutional or sustainability-centered pursuits. Evolved reward systems and leaderships modes that support collective and institution-level effort and innovations prioritizing community and sustainability require new leadership models. Innovative leadership models that transcend traditional gatekeeping are needed and four leadership modes to support innovation and collective efforts are discussed, including shared leadership that draws on distributed contributions of multiple individuals; creative or innovative leadership that requires risk-taking, experimentation, and experiential learning; qualitative leadership that is data-driven and includes evidence-based innovation; and, dynamic leadership based on demonstrated agility and ability to traverse different spaces using diverse modes of doing and thinking. Progressive leaders can move in and out of these modes in response to ecosystem needs, demands, and changes through the use of design thinking and initiatives to support innovation and sustainability in higher education. Success in evolved leadership approaches, including centering sustainability goals that impact institutions themselves and communities in which they exist, require aligning reformed leadership goals and practices with funding models and reward systems, as well as policies and institutional change strategies.

## Introduction

Recent global concerns around the COVID-19 pandemic and longstanding social justice issues including the impacts of institutionalized racism and bias, have highlighted significant short-comings in our approaches to leadership selection as well as culture change and management across the academic landscape [1–3]. Common or more traditional forms of leadership in academia can tend towards ‘command and control’ models rather than collective or collaborative forms of leadership [4]. Prior discussion of the higher education leadership continuum recognizes a progression from transactional management approaches that align with command and control, to administration that is more relational, mission-driven, and focused on development to ‘true leadership’ that is positioned as transformational and vision-driven [5]. Even prior to the current challenging time, many academic leaders ascended to leadership roles based on success in research, scholarship, and managerial approaches and often in the absence of formal leadership training or preparation [2, 3, 6, 7]. Sustainable and needed change in higher education must occur throughout multiple levels in the current ecosystem. Further, the ability or inability to pursue change successfully depends on ecosystem culture and agility as well as leadership expectations and practices or lack thereof.

**Fig. 1:**
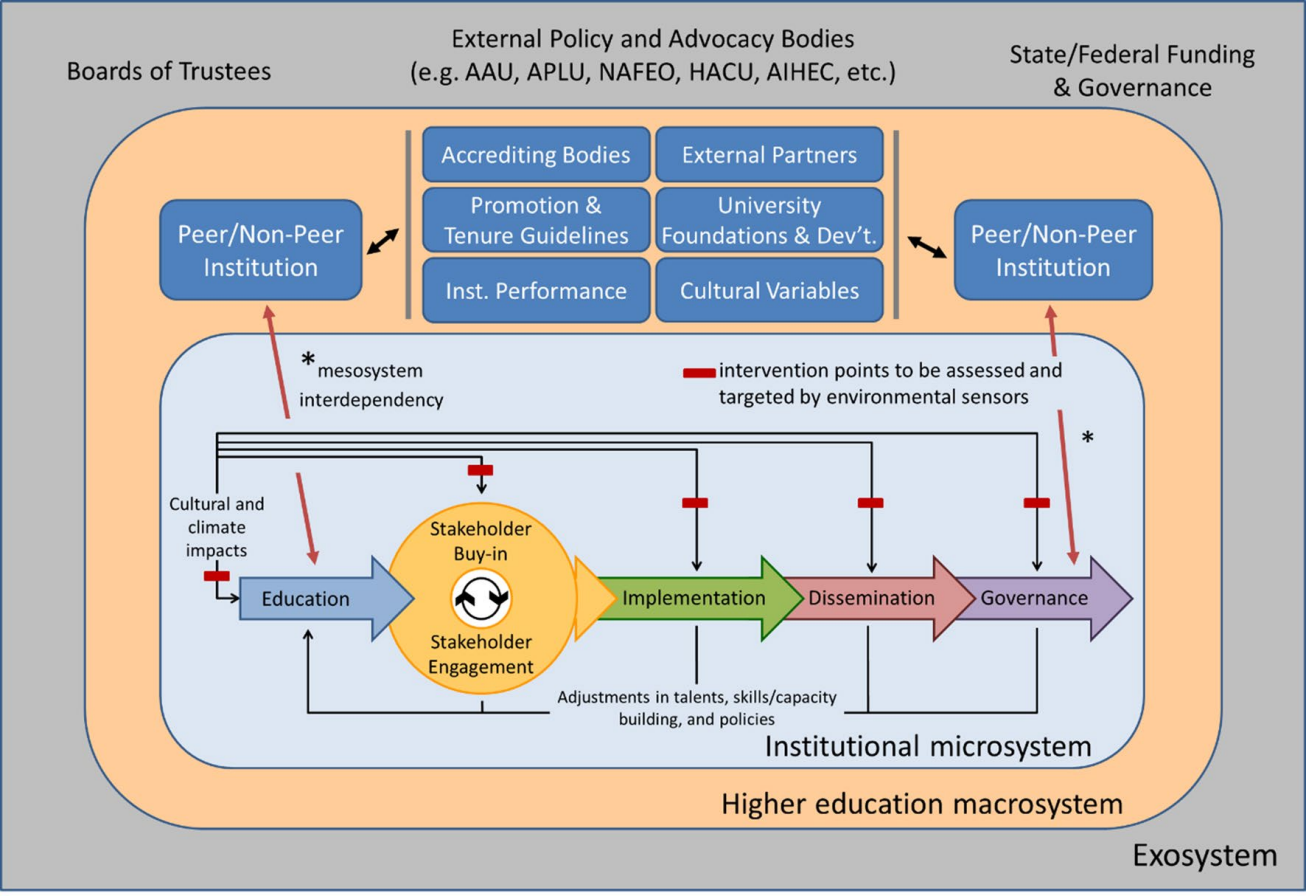
Academic institutional-change centered ecosystem model. *Institutional microsystem* (in light blue box): Local institutions must focus on institutional transformation by facilitating and integrating local education, followed by buy-in and engagement of stakeholders, subsequently followed by moving to the action of implementation, dissemination, and promoting sustainable change through governance. Red bars represent environmental sensors who function to assess change and promote interventions for change and innovation (adapted from Montgomery [32]). Feedback from the paradigm supports adjustments in talent, skills/capacity building, ad policies, the latter of which determines how change, rather than stagnation or status quo, occurs in a system. *Higher education macrosystem* (in light orange box): Efforts at a local institution are impacted through mesosystem interdependencies (red arrows traversing local institution ecosystem and higher education ecosystem) including evaluations of peer and non-peer institutions (as denoted by traditional hierarchical frameworks typified in the Carnegie Classification System for representing institutional diversity in types). Additionally, the macrosystem is impacted by factors and entities including accrediting bodies, promotion and tenure guidelines, institutional performance metrics, external partners (e.g., industry, non-profits, etc.), university foundations and development/advancement, and cultural variables. *Exosystem* entities (in gray box): The higher education ecosystem as a whole is impacted by governing boards (e.g., Boards of Trustees), external policy and advocacy bodies, such as Association of American Universities (AAU), Association of Public and Land-Grant Universities (APLU), National Association for Equal Opportunity in Higher Education (NAFEO), Hispanic Association of Colleges and Universities (HACU), and American Indian Higher Education Consortium (AIHEC), among others, and state and federal funding agencies and governance bodies

When focused on ecosystem-based approaches and ecosystem-wide outcomes, it is critical to recognize that an ecosystem consists of a network of structures, environmental contexts (e.g., microsystems, mesosystems, and exosystems according to Bronfenbrenner [8]), policies and practices, and the people operating therein (Fig. 1). Microsystems consist of the direct individual interactions between faculty, staff, leaders, or individual stakeholders, including for example departments, academic programs, and colleges within higher education or community or disciplinary societies outside; mesosystems are comprised of interactions between microsystems, including interactions between a department and disciplinary society or between two institutions; macrosystems are the larger cultural systems and societal beliefs about the system in which individuals exist; and, exosystems are the informal social structures outside of the core ecosystems and processes that influence individuals functioning within systems [8, 9]. Both the culture and composition of an ecosystem underlie its performance; and culture is built and maintained by people and involves the norms and enactment of policies [10]. Multi-level susceptibility to risks and failures can occur in an ecosystem simply due to its complexity [11].

Part of promoting access, accountability, and needed change requires that leaders assess ecosystems to identify, understand, and navigate cultural mistrust vs. cultural alignment [12]. Leaders need recognize the importance of appropriately assessing culture, prevalence of siloes, and networks, as well as how they interface in order to leverage them in support of overcoming obstacles and barriers. Doing so will facilitate a promotion of trust and progress towards ecosystem transformation.

Leaders (particularly executive leaders) are, or should be, essential purveyors in stewardship of ecosystems [1]. Effective ecosystem leadership and sustainability of ecosystems require careful assessment (inclusive of network and cultural analyses) of microsystems, but relative to the effectiveness and connectivity of macrosystems responsible for driving mission and vision activities. Such ecosystems-focused leadership supports the identification and development of talent pools as well as mitigation of local threats or risks required to obtain a true picture of the health and sustainability of the larger ecosystem.

Supporting needed change in systems to move towards sustainability requires recognizing and navigating local microsystem-level issues, as well as negotiating interactions with cultural inertia and inefficient leadership, which are linked to resource allocations (human, financial, etc.) and accountability. Distinct modes of leadership may be required at different stages of a cultural change or transformation process to facilitate forward progress and sustainability [13, 14].

## Issues with current academic leadership models for promoting change

In discussing needed ecological interventions related to issues such as environmental climate change, Lent [15] indicated that “we need to forge a new era for humanity—one that is defined, at its deepest level, by a transformation in the way we make sense of the world, and a concomitant revolution in our values, goals, and collective behavior.” We argue that this is also true of interventions in leadership needed for cultural climate change—both inside the academy and far beyond. Barnett [16] advanced the theory of the “ecological university” which positions an institution as aware of and operating from an understanding of global interconnectedness at the interface of institutions with the world. Thus, we must be more specific about goals, understanding the development and roles of policies in these spaces, and the impact of policies on individual versus institutional strategic goals and objectives, including in a larger global context. In higher education, policies are generally directed towards individuals and individual accomplishments, so how can we appropriately account for successfully promoting collective behavior and achievement of institutional objectives as well as understanding the interface with communities and the larger global society beyond?

The individual versus institutional achievement conundrum represents a major point of cognitive and functional dissonance and constitutes a significant leadership challenge [17]. The policies and expectations related to measurable scholarly output—individual scholarly or creative works—are pervasive though we in higher education increasingly purport interest in and commitment to interdisciplinary and collaborative work. Yet, when it is time to assess the progress of individuals, leaders still attempt to break down scholarly effort, even for purportedly highly interdisciplinary and team-based scholarly endeavors, into units of “individual credit” to allow individuals to have a sense of reward and to support our efforts to quantify, often erroneously, individual contributions [18].

Leaders indeed are often selected for their commitment to enact traditional and/or transactional measures of assessing scholarly contribution and impact [1] and to pursue traditional measures of prestige and relative institutional standing [2]. Leaders frequently default to the perspective that we do not know how to deal with interdisciplinary, transdisciplinary, or team-based efforts and scholarly outputs and, thus, become entrenched in assessment of individual work, attempts to calculate individual contributions to collaborative effort, and utilization of status quo or standing policies [19, 20]. Ultimately, flawed key performance indicators can be used that represent poor alignment between process, policy and expected or perceived outcomes. In this regard, managerial leaders often contribute to the problem of a general failure in higher education to evolve policies that would use both evidence of and innovation in approaches, i.e., what we refer to here as evidence-based innovation, or the use of new parameters and metrics for assessing evolved frameworks of scholarship based on non-standard outputs and integrated collaborative and interdisciplinary efforts. Thus, change and transformation are stymied by a lack of will and a dearth of local creativity on the part of leaders to move towards the development and utilization of policies that would recognize and reward collective behavior.

Leaders want the benefit of the creative, innovative outcomes that come from cutting-edge collaborative or collective work but have not advanced the transformation of evaluative systems of assessment and reward that recognize that work [21–23]. Recent recommendations related to developing metrics as well as narrative evidence to support recognizing and rewarding significant innovation and entrepreneurial impact as a part of core review and reward processes that emerged as part of the outcomes of the recent Promotion and Tenure–Innovation and Entrepreneurship (PTIE) Summit represent major first steps in addressing these issues related to creative evaluation of innovations in scholarly recognition and reward [24]. Failure to address the dissonance in expressed commitment to innovation with the persistence of traditional individual success measures represents a pervasive leadership deficit. We propose that evidence-based innovation is critically needed in terms of leaders supporting, assessing, and rewarding collective effort and behavior to mitigate this deficit. Furthermore, these innovations are often linked to the mission of a university and commitments to improving the human condition that links to sustainability.

Part of the failure to innovate evaluation and reward policies and practices is related to a culture of competition and imitation in higher education. This has been frequently described in business circles as “swimming in a sea of sameness” [25]. Such a state often represents institutions trying to emulate each other’s outcomes and achievements; rather than promoting activities based on careful, focused assessments of their own intrinsic institutional talents, strengths, and cultures. Additionally, universities rely on competition for financial solvency; yet this can challenge institutional commitments to sustainability that support human health and well-being as central factors in institutional work [4].

## Innovative models of academic leadership to address evolving educational needs

**Fig. 2:**
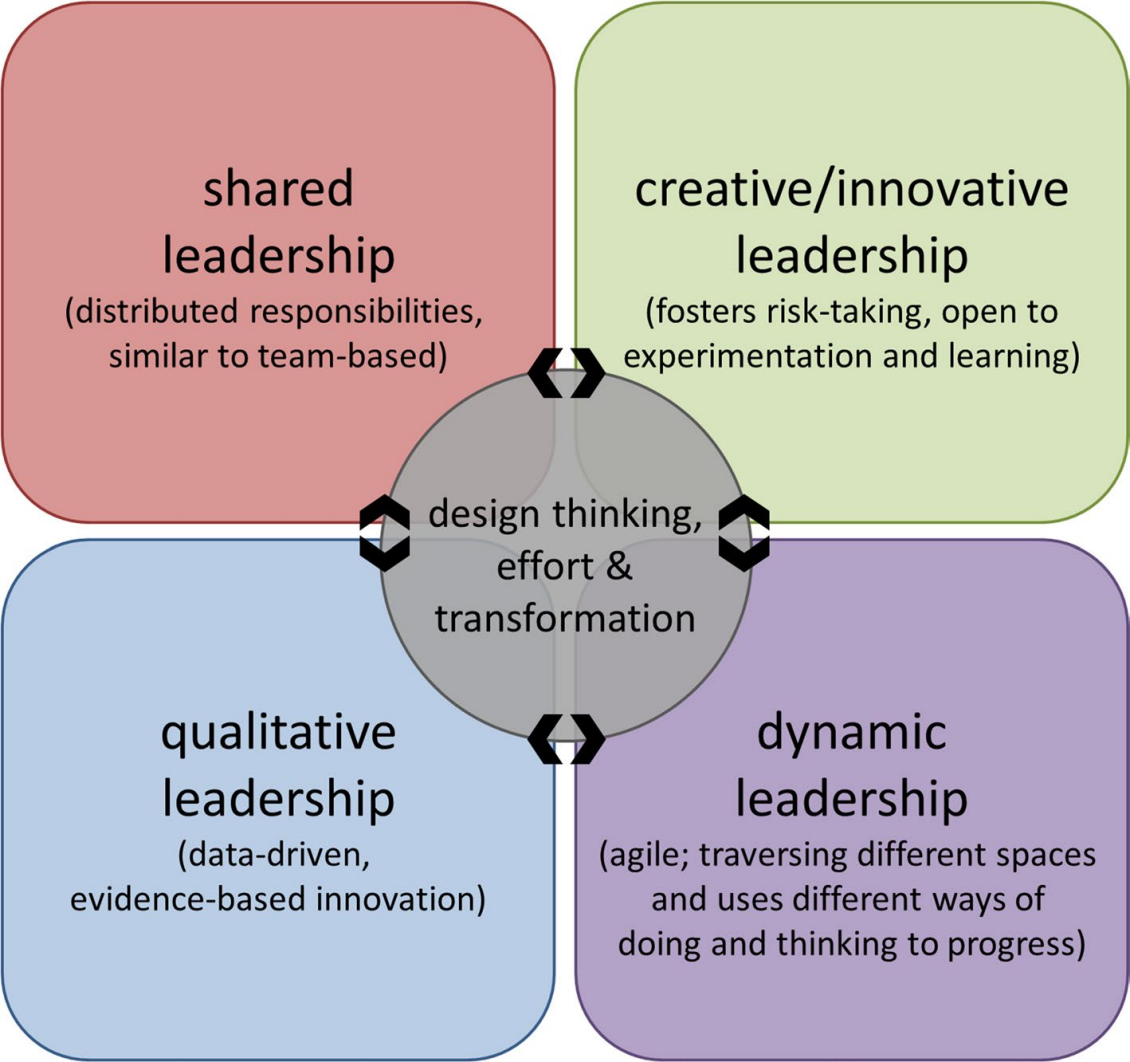
Innovative model for academic leadership. New modes for academic leadership that transcend traditional gatekeeping modes of leading that maintain status quo are needed and four are proposed here, including shared leadership, creative or innovative leadership, qualitative leadership, and dynamic leadership. Progressive leaders (represented by the center gray circle with bidirectional arrowheads) are able to move in and out of these four modes in response to ecosystem needs, demands, and changes through using design thinking and initiating effort towards ecosystem transformation. Decision-making and analytics informed by real-time data acquisition and environmental dynamics can dictate the rate and timing of pivotal shifts through the respective leadership modes

We have previously argued that new “models of leadership are needed to reimagine higher education and to bring this reimagination effectively and sustainably to fruition” [2]. We proposed that pivoting from managerial, quantitative, and individualistic leadership frameworks will require testing the efficacy of new leadership paradigms. Here, we highlight four models of leadership as relevant for promoting culture change and sustainability in higher education. These models include the following: shared leadership, creative or innovative leadership, qualitative leadership, and dynamic leadership (Fig. 2). These leadership modes are not positioned as mutually exclusive as the characteristics most beneficial could depend heavily on ecosystem variables and culture in which one is leading.

Shared leadership includes leadership that draws on distributed responsibilities across multiple individuals in an ecosystem. Shared leadership, thus, is similar to team-based leadership with input from a team of individuals [26, 27]. Team-based leadership models have been associated with higher levels of group performance [28].

Creative or innovative leadership, sometimes also referred to as entrepreneurial leadership, is typically poised to respond to crisis and disruption, and to address circumstances out of the norm [29–31]. Recent examples include leadership responses to the COVID-19 surge and in addressing systemic racism—situations in which leaders needed to come up with thoughtful, creative, and innovative responses due to major ecosystem disruptions [1, 32]. Creative/innovative leadership fosters risk-taking and effective leaders operating in this frame are open to experimentation and learning.

Qualitative leadership is largely based on decision makers who draw on institutional data and analytics capabilities [33]. The goal of qualitative leadership is to “enhance the quality (as opposed to increasing or growing the quantity) of something” [34]. These leaders, however, often use data or evidence paired with innovation or novel experimentation to adapt test models to a local context and culture. Such evidence-based innovation is critical to ensure adapting leadership to a local ecosystem, with full knowledge of macrosystem and exosystem level factors that influence the local institutional operations and productivity.

Dynamic leadership is based on a journey where an agile leader walks a path in and out of different spaces within an ecosystem, drawing on diverse talents and perspectives, as well as ways of thinking and doing [35, 36]. These leaders so function to stay committed to progress, while simultaneously making the most of the leader’s inherent strength which is a hallmark of agility. Dynamic leaders are good communicators and understand that their success depends on cultivating relationships and understanding context [37].

Across all these progressive leadership modes, centering mission and vision is critically important. Mission-centered management and leadership is a viable solution in which every action step is aligned with a leader’s (or institution’s) mission and vision and drives decision-making and strategic investments. Responsibility-centered management is currently prevalent in many higher education ecosystems; yet it can be problematic because it is common in this framework for people to simply follow lines of responsibilities in a job description or to competitively accrue resources, rather than being inspired by or rewarded for collective effort, innovation and collaborative entrepreneurial thinking [38]. Visionary and forward-looking individuals may not be attracted to such systems.

In dynamic ecosystems, effective leaders may have to draw on all four leadership modes at different times and in response to distinct ecosystem demands. Leaders who can function in this way can draw on the synergy of the four leadership modes presented. These are individuals able to lead in dynamic systems with rapid change and by oscillating between leadership modes as needed to stay current, relevant, and agile. Enacting such a synergistic leadership framework will require transforming “the process for selecting leaders to guide universities toward an equitable and sustainable future” [2].

Some of the limits with traditional leadership models that espouse commitments to innovative and interdisciplinary scholarly efforts yet continue to employ traditional metrics of individual success and advancement are clear. Barriers to evolving systems of review and reward to match championed commitments to innovation and collaboration include limitations in determining how to build collaborative and integrative academic ecosystems with “transient widgets” in the context of hierarchical power structures and traditional sociocultural dynamics of leaders, as well as challenges related to economic and technology implications and differences between distinct institution types. We must address these cultural issues related to leadership to sustainably engage new leadership models such as those described above.

## Implications of new leadership models

There are many levers to pull to promote needed changes and innovations in leadership to support transitions to frameworks that recognize collective talent, aptly review and reward collective and interdisciplinary contributions, and develop shared vision about enacting these new modes of scholarship and leadership support. First is to recognize that leadership is about influence, and that there is a need for leaders to be able to engender confidence and hope, to demonstrate courage and thoughtful engagement, to drive buy-in that supports change, impact and sustainability through collective effort and capabilities, and to harness collective potential. Such leadership efforts must align with abilities or capabilities within an ecosystem to track dynamics closely and to result in leaders being effective in making critical and timely decisions.

So, what does it take to develop an “appropriate” leadership mindset that would support evidence-based innovation in context and ultimately drive ecosystem transformation? This would require multiple, simultaneous considerations, including how to measure and evaluate meaningful leadership and ecosystem progress and impact, which depend upon the identification of variables and environmental characteristics as key performance indicators [39]; how to support, evaluate and reward both individual effort and contributions to collective advancement; how to facilitate shared understanding of expected outcomes; how to recognize and mitigate challenges and limitations of the processes we use to facilitate evolving them; and, how to innovate to develop evolved or novel models of assessment to dissipate tensions between static, status quo individual success paradigms compared to those that recognize coherence or collaborative team science. Also required is an ability for leaders and others in an ecosystem to embrace the unknown and uncertainty, as well as the role of educational leadership in enabling and facilitating sustainability.

One of the first issues that must be addressed is that progressive collaboration and evolved evaluation processes require a sense of trust [40, 41]. Trust is a cornerstone of success for thriving ecosystems. It must be embedded in all processes and practices—the very platform in which you operate, including addressing systemic issues to promote cultivating relationships across disparate units and places on campus. If the goal is for an ecosystem to thrive and provide solutions for everyone, trust must be cultivated and elevated. Lack of trust leads to ineffective networks and incoherent interactions which promote dysfunction, as well as compromise growth and progress, which is not ideal for sustainability or ecosystem transformation.

## Conclusions

Leaders should demonstrate and/or provide evidence of consistency in routinely being creative, innovative, and agile. We generally define or recognize agility in assessing leaders’ responsibility to address challenges and incidences that lead to instability. Yet, progressive and sustainable ecosystems will select and reward leaders who are open to novel perspectives and are constantly learning. Such leaders demonstrate dynamic abilities to assess, function creatively, draw on data from the environment to support decision making, drive process improvements, and develop strategies to build momentum towards progress through reflection, trust-building, and broad interactions/engagements across the ecosystem. Agile leaders in such contexts lead innovation in a dynamic environment to meet needs of students, staff, and other stakeholders in an ecosystem. Context-based leadership models that respond with agility and with dynamism have great relevance and aptitude for driving sustainability-centered initiatives [16].

Future leaders, if they are learning leaders, can develop knowledge capital to network and “think together” with other leaders as well as subordinates to position institutions for transformation across the higher education spectrum of institutions and ecosystem to leverage knowledge gained from others in similar positions, but also to support development of distinct perspectives about the ecosystems that they are managing. Such an approach exemplifies evidence-based innovations in context and can lead to new leadership and governance strategies, as well as approaches for transformative change and sustainability.

## Data Availability

Not applicable.
